# Reconstructive Goals in Arm and Elbow Defects Treated with the Pedicled Latissimus Dorsi Flap

**DOI:** 10.3390/jpm16050260

**Published:** 2026-05-13

**Authors:** Ömer Kokaçya, Umut Dalgıç, Abdullah Arslan, İbrahim Tabakan, Gazi Kutalmış Yaprak, Ahmet Cemil Dalay, Erol Kesiktaş

**Affiliations:** 1 Department of Plastic Reconstructive and Aesthetic Surgery, Faculty of Medicine, Cukurova University, Adana 01330, Türkiye; 2 Private Practice, Adana 01140, Türkiye

**Keywords:** latissimus dorsi flap, upper extremity reconstruction, elbow flexion, electrical burns, limb salvage, personalized reconstructive surgery, individualized treatment planning

## Abstract

**Background/Objectives:** Reconstruction of complex soft-tissue defects of the arm and elbow remains challenging because of exposed neurovascular structures, wide joint mobility, and the need to preserve function. The pedicled latissimus dorsi (LD) flap remains a valuable option, particularly when recipient vessels are compromised or functional restoration is required. Given the heterogeneity of these injuries, treatment must be individualized according to each patient’s defect characteristics, functional demands, and rehabilitation goals, reflecting personalized medicine principles. This study evaluated the indications and outcomes of pedicled LD flap transfer in arm and elbow defects. **Methods:** All consecutive patients who underwent pedicled LD flap reconstruction for upper extremity soft-tissue defects at our institution (January 2015–January 2025) were retrospectively reviewed. Demographic data, defect etiology, flap type, reconstructive goals, complications, and functional outcomes were analyzed. **Results:** Twenty-six patients were included (mean age 28.5 ± 7.6 years; 84.6% male). Electrical burns were the predominant etiology (92.3%). A musculocutaneous flap was used in 22 patients (84.6%) and a muscle-only flap in 4 (15.4%); supplementary split-thickness skin grafting was required in 17 (65.4%). Reconstructive goals included elbow flexion restoration (±neurovascular repair and soft-tissue coverage) in 12 patients (46.2%) and humeral stump preservation for prosthetic use in 14 (53.8%). No total flap loss occurred. Complications included partial necrosis in 1 patient (3.8%), donor-site seroma in 3 (11.5%), wound dehiscence in 2 (7.7%), and recipient-site hematoma in 1 (3.8%). No patient required amputation or shoulder disarticulation. **Conclusions:** The pedicled LD flap is a reliable option for complex arm and elbow defects. By tailoring flap design, nerve management, and rehabilitation to individual patient needs, this approach exemplifies personalized reconstructive planning in upper extremity trauma.

## 1. Introduction

Upper extremity reconstruction, particularly defects involving the elbow and arm, presents distinct reconstructive considerations in plastic surgery. The thin skin and subcutaneous tissues in this region, the wide range of joint motion, and the superficial course of vital neurovascular structures are the main factors that shape the reconstructive approach. Extensive soft tissue defects resulting from traumatic injuries, electrical burns, oncologic resections, and chronic infections may lead not only to functional impairment but also to a high risk of infection and complications that threaten limb integrity [1,2]. Therefore, providing reliable, durable, and functional soft tissue coverage remains one of the primary goals of reconstructive surgery [2].

The pedicled latissimus dorsi flap was first described by Tansini in 1906 for breast reconstruction and began to be used in upper extremity reconstruction in the mid-20th century [3]. Since then, numerous studies have demonstrated its reliability, particularly in the coverage of large and complex defects [4,5]. The flap can be harvested either as a muscle or musculocutaneous flap, allowing it to adapt to different types of defects. Moreover, when used as a functional transfer, it can restore elbow flexion or extension by substituting the function of the biceps or triceps muscles [5].

The latissimus dorsi (LD) muscle is classified as a type V muscle flap according to the Mathes and Nahai classification, possessing a dominant vascular pedicle as well as secondary segmental pedicles. This powerful muscle, supplied by the thoracodorsal artery and vein, is considered one of the workhorse flaps in reconstructive surgery due to its large surface area and long vascular pedicle. The thoracodorsal pedicle has an average length of approximately 6–9 cm from its origin at the subscapular artery to the entry point into the muscle, although effective pedicle length can be further extended by dissecting along the subscapular axis [6]. It has been reported that the thoracodorsal artery divides into two (55%) or three (45%) branches within the muscle [6]. However, the main limitation of the pedicled LD flap is its restricted arc of rotation, which confines its reliable reach to the arm and elbow region; defects distal to the elbow typically require free tissue transfer.

Advances in microsurgical techniques and the widespread use of free tissue transfer have not diminished the importance of pedicled flaps. On the contrary, the pedicled latissimus dorsi flap remains an important reconstructive option in cases where free flap transfer is not feasible, when recipient vessels are unreliable, or in emergency trauma settings and contaminated defects [7]. However, the wide spectrum of injury patterns encountered in clinical practice—ranging from isolated soft-tissue loss to composite defects involving bone, vessels, and nerves—renders a uniform reconstructive strategy inadequate. Treatment planning must therefore be individualized, taking into account each patient’s specific defect characteristics, functional demands, and rehabilitation potential, in line with the principles of personalized medicine in reconstructive surgery [8]. In this study, the role of the pedicled latissimus dorsi flap in arm and elbow defects, its indications for use, and its clinical outcomes were evaluated with particular emphasis on how reconstructive goals were tailored to individual patient profiles.

## 2. Materials and Methods

### 2.1. Study Design and Patient Selection

In this retrospective study, we reviewed all consecutive patients with upper extremity soft-tissue defects who underwent reconstruction with pedicled latissimus dorsi flaps at our institution between January 2015 and January 2025. Inclusion criteria were (1) upper extremity soft-tissue defects requiring flap reconstruction, (2) use of a pedicled LD flap, and (3) a minimum follow-up of 12 months. The follow-up duration ranged from 15 months to 6 years. Patients with incomplete medical records were excluded. A total of 26 patients met the study criteria and were included in the analysis.

### 2.2. Data Collection and Outcome Measures

Medical records were retrospectively reviewed, and the following variables were analyzed: demographic characteristics, defect etiology, flap type (musculocutaneous or muscle), additional procedures performed in the same surgical session for the repair of associated vascular or nerve injuries (if present), reconstruction goals, and postoperative complications. Postoperative complications were defined as any flap-related or donor-site adverse events occurring during the follow-up period, including partial or total flap necrosis, wound dehiscence, seroma, hematoma, and the need for amputation. Complications were assessed through inpatient evaluations and routine outpatient follow-up visits based on clinical examination. Reconstruction goals were defined as coverage of exposed neurovascular structures, repair of major vessels and nerves, restoration of elbow flexion, and preservation of the humeral stump to allow future prosthetic use.

### 2.3. Operative Technique

All procedures were performed under general anesthesia with the patient in the lateral decubitus position, with the ipsilateral upper extremity draped free to allow intraoperative positioning and inset. The skin paddle, when planned, was marked over the posterior axillary fold according to the defect requirements. The latissimus dorsi muscle was exposed through a posterior axillary incision. With the arm positioned in abduction, forward flexion, and external rotation to stretch the muscle, the lateral half of the muscle was harvested to match the size of the biceps, as this portion has been shown to be adequate for elbow flexion restoration while minimizing donor-site morbidity [9,10]. The thoracodorsal artery, vein, and nerve were identified and carefully preserved as they entered the muscle approximately 10–12 cm from the axilla. A unipolar transfer technique was utilized in all patients requiring elbow flexion restoration: the origin of the latissimus dorsi was released from the thoracolumbar fascia and paraspinal attachments corresponding to the harvested segment, while the humeral insertion was maintained intact. This approach avoided manipulation of the neurovascular pedicle, thereby reducing the risk of pedicle compromise, hematoma, and thoracodorsal nerve injury [9]. The mobilized muscle was tubularized and passed through a subcutaneous tunnel to the anterior aspect of the arm through a separate antecubital incision. The distal end of the muscle was secured to the distal biceps tendon when a suitable biceps remnant was present; in cases without a viable biceps tendon, a pseudotendon was created from the epimysium and fibrous remnants of the latissimus dorsi attachments and secured to the radius through transosseous sutures, as described by Martin et al. [11]. Tension was set with the elbow in extension to achieve an appropriate resting muscle length. The thoracodorsal nerve was preserved in all patients for whom restoration of elbow flexion was the primary objective and transected in all patients who underwent humeral stump reconstruction. The choice between musculocutaneous and muscle-only flap design was based on the availability of donor-site skin and whether simultaneous soft-tissue coverage was required. A muscle-only flap was selected when the overlying donor-site skin was unsuitable for inclusion due to chronic burn wounds or scarring and when the recipient site had intact soft tissue. When the defect area exceeded the coverage provided by the skin paddle of a musculocutaneous flap, an additional split-thickness skin graft was applied over the exposed muscle to complete the coverage. In cases with vascular and nerve injuries, the brachial artery was repaired using a saphenous vein graft, while the median nerve was reconstructed using a sural nerve graft.

Particular attention was paid to donor-site closure with the aim of minimizing seroma formation. Electrocautery was used at the lowest effective setting, and the dissection field was intermittently irrigated with saline during its use to reduce thermal injury to the surrounding tissues. Following meticulous hemostasis, the subcutaneous skin flaps were secured to the underlying chest wall using multiple interrupted absorbable quilting sutures placed in rows to obliterate the dead space. A closed-suction drain was placed in the donor cavity and maintained until drainage output fell below 30 mL per day. The skin was closed in two layers.

### 2.4. Postoperative Rehabilitation

Postoperative rehabilitation was individualized according to each patient’s reconstruction goal and functional capacity [10]. In patients undergoing LD transfer for elbow flexion restoration, the elbow was immobilized in 90 degrees of flexion for 3–4 weeks, followed by gradual passive and active-assisted range of motion exercises. Active elbow flexion exercises were initiated at 6 weeks postoperatively and progressively intensified under the supervision of a physiotherapist. In patients receiving coverage-only flaps or stump preservation, early mobilization of the shoulder was encouraged to minimize donor-site stiffness, and supervised physiotherapy was initiated as wound healing permitted.

### 2.5. Statistical Analysis

Descriptive statistics were used to summarize the data. Continuous variables are expressed as means (± standard deviation) and ranges, while categorical variables are presented as numbers and percentages.

## 3. Results

A total of 26 patients were included in the study. The majority of the patients were male (22 patients, 84.6%), while 4 patients (15.4%) were female. The mean age was 28.5 ± 7.6 years (range: 8–35 years). The follow-up duration ranged from 15 months to 6 years. Electrical burns were the most common cause of injury, accounting for 24 cases (92.3%), whereas traffic accidents were responsible for 2 cases (7.7%). The demographic characteristics and etiological distribution of the patients are summarized in Table 1. In 22 patients (84.6%), a musculocutaneous flap was used; the skin paddle was inset over critical exposed structures, and in 14 of these cases (63.6% of musculocutaneous flaps), an additional split-thickness skin graft was required to cover the residual defect area not addressed by the skin paddle, while in the remaining 8 cases, the skin paddle alone was sufficient for coverage. In the other 4 patients (15.4%), a muscle-only flap was harvested. In 3 of these cases, the overlying donor-site skin was not suitable for inclusion in the flap due to burn wounds or scarring from the original injury, and the transferred muscle was resurfaced with a split-thickness skin graft. In 1 patient, who underwent delayed reconstruction for elbow flexion restoration only, the recipient-site skin envelope was intact, and no additional coverage was required; therefore, a muscle-only transfer without skin grafting was performed. Overall, a split-thickness skin graft was used as part of the reconstruction in 17 patients (65.4%).

### 3.1. Reconstruction Goals

Reconstruction goals varied according to the extent of injury and associated functional deficits (Table 2). Reconstruction goals were categorized into two main groups: (1) elbow flexion restoration (± neurovascular repair and soft-tissue coverage) and (2) humeral stump preservation for prosthetic rehabilitation.

**Figure 1:**
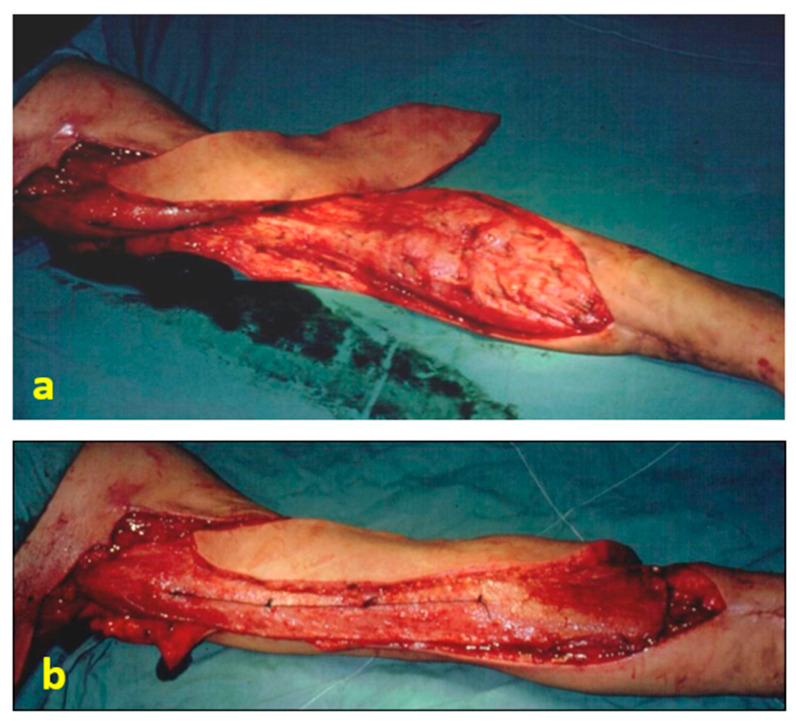
Intraoperative view: (**a**) Flap transferred to the antecubital region. (**b**) After flap inset, with the flap positioned to mimic the biceps brachii muscle.

**Figure 2:**
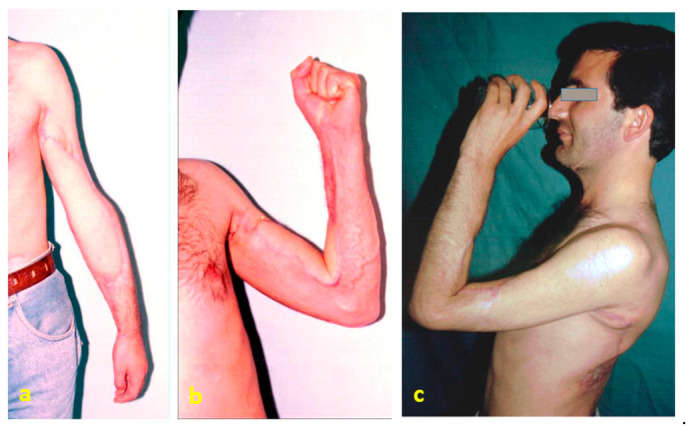
Postoperative 6-month view: (**a**) anterior view; (**b**) elbow in flexion. (**c**) The patient is able to bring a glass of water to the mouth without assistance.

Combined brachial artery and median nerve repair with soft-tissue coverage and restoration of elbow flexion was performed in 2 patients (7.7%). Brachial artery repair with soft-tissue coverage and restoration of elbow flexion was performed in 2 patients (7.7%). In these four patients who underwent brachial artery repair using saphenous vein grafts, vascular continuity was successfully restored, adequate distal extremity perfusion was achieved, and none required additional vascular intervention after reconstruction. Soft-tissue coverage with restoration of elbow flexion was achieved in 7 patients (26.9%). In one patient (3.8%) who underwent delayed reconstruction, restoration of elbow flexion alone was the primary objective (Figure 1 and Figure 2).

**Figure 3:**
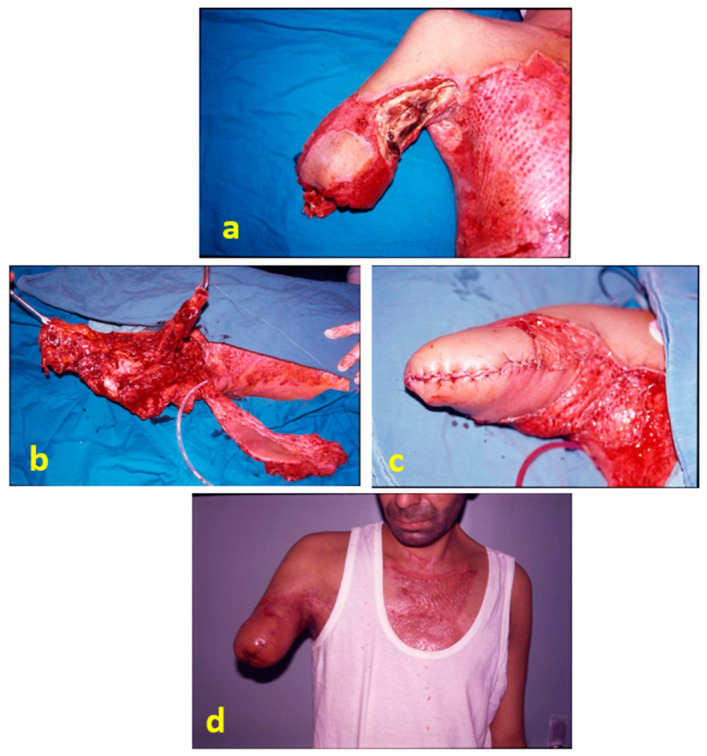
A patient who underwent a transhumeral amputation due to an electrical burn: (**a**) soft-tissue defect at the stump; (**b**) flap transferred to the recipient site after debridement; (**c**) after flap inset; (**d**) postoperative view at 3 months.

In 14 patients (53.8%) who had already undergone transhumeral amputation and still had soft tissue defects at the stump, the main reconstructive goal was preservation of the humeral stump to allow future prosthetic use and to prevent the need for shoulder disarticulation. During the follow-up period, none of these patients required a more proximal amputation or shoulder disarticulation. A representative case is shown in Figure 3.

### 3.2. Functional Outcomes

Functional status was evaluated through clinical observation during outpatient follow-up visits. Among the 12 patients in whom restoration of elbow flexion was a primary reconstructive objective, 9 patients (75%) achieved a functional range of active elbow flexion estimated at 90° or greater at the final follow-up. These patients were able to independently perform essential activities of daily living, including bringing a glass of water to the mouth, self-feeding, and grooming activities such as combing the hair (Figure 2). The remaining 3 patients demonstrated improved but more limited active elbow flexion, estimated at approximately 60–80°, which represented a meaningful improvement from their preoperative status and allowed partial independence in daily tasks, although full overhead reach and some bimanual activities remained limited. Antigravity elbow flexion strength was achieved in 10 of 12 patients (83.3%).

In the 14 patients whose primary reconstructive goal was humeral stump preservation for prosthetic use, all patients maintained an amputation level compatible with prosthetic fitting throughout the follow-up period, and none required revision to a more proximal amputation level or shoulder disarticulation.

### 3.3. Complications

Total flap loss was not observed in any of the patients. Partial skin necrosis developed in one patient who underwent a musculocutaneous flap and resolved with secondary healing. Donor site complications included wound dehiscence in 2 patients (7.7%) and seroma in 3 patients (11.5%), while no donor site hematoma was observed. Donor-site wound dehiscence was managed with secondary suturing, while donor-site seroma was managed with repeated transcutaneous aspiration. A recipient site hematoma occurred in 1 patient (3.8%) and was managed with reoperation to achieve hemostasis. Importantly, none of the patients required amputation, stump revision, or shoulder disarticulation (Table 3).

## 4. Discussion

The upper extremity is particularly vulnerable to trauma owing to its high degree of mobility and indispensable role in daily activities. High-energy injuries, in particular, often result in complex defects characterized by extensive soft-tissue loss, associated bone and joint destruction, and damage to major neurovascular structures, ultimately leading to substantial functional impairment. Although the distal upper extremity is more frequently exposed to trauma, injuries involving the elbow and arm also present significant reconstructive challenges. In this region, critical neurovascular structures such as the brachial artery and the median, radial, and ulnar nerves follow a relatively superficial course, rendering them particularly vulnerable to high-energy trauma. The ulnar nerve, in particular, is susceptible due to its superficial course at the medial epicondyle, and its injury can result in significant loss of intrinsic hand function, grip strength, and sensation of the ulnar digits [12,13]. Consequently, these structures are often either directly injured or become exposed secondary to extensive soft-tissue defects. Furthermore, such injuries may lead to loss or severe dysfunction of the arm flexor muscles, primarily the biceps brachii, the primary flexor of the elbow, resulting in substantial impairment of upper limb function. In more severe cases, particularly in injuries located closer to the shoulder, limb salvage may not be feasible, and amputation can become inevitable [14,15,16].

The heterogeneity of upper extremity defects—in terms of etiology, anatomical location, tissue involvement, and functional impact—necessitates an individualized approach to reconstructive planning. A standardized algorithm cannot adequately address the wide spectrum of clinical scenarios encountered in practice. In our cohort, reconstructive goals ranged from simple soft-tissue coverage to complex composite reconstruction involving simultaneous vascular and nerve repair with functional muscle transfer and from elbow flexion restoration to humeral stump preservation for prosthetic use. Each of these goals required a distinct surgical strategy, flap design, and postoperative rehabilitation protocol. This patient-centered, goal-directed framework exemplifies the application of personalized medicine principles in reconstructive surgery, where clinical decisions are driven by the individual patient’s anatomical, functional, and psychosocial profile rather than by a rigid protocol [17].

Reconstructive options vary depending on the size and complexity of the defect. In superficial defects, secondary healing or skin grafting may be sufficient; however, these methods are inadequate in cases with exposed bone, tendon, or neurovascular structures. In superficial soft-tissue defects of the arm and elbow where the underlying muscular architecture is preserved, perforator-based flaps and local fasciocutaneous flaps offer excellent reconstructive solutions without the need to sacrifice a functional muscle such as the latissimus dorsi. Pinto et al. reviewed the reconstructive armamentarium for upper limb defects and described a range of perforator-based propeller flaps around the elbow and distal arm, each based on named perforators located just proximal to the elbow joint: the inferior ulnar collateral artery perforator (IUCAP), superior ulnar collateral artery perforator (SUCAP), brachial artery perforator (BAP), radial recurrent artery perforator (RRAP), and radial collateral artery perforator (RCAP) [18]. Flaps based on IUCAP, SUCAP, and BAP lie on the medial aspect of the arm and are suitable for anterior, medial, and posterior elbow defects, providing thin, pliable, hairless skin with a less visible medial donor-site scar. RCAP- and RRAP-based flaps are harvested from the lateral arm and are indicated for lateral elbow defects. The lateral arm flap, based on perforators of the posterior branch of the radial collateral artery, can be used either as a reverse pedicled flap centered on the radial recurrent artery or as a free antegrade flap and is particularly useful for posterior elbow coverage [18]. The thoracodorsal artery perforator (TDAP) flap, described as the perforator evolution of the LD flap, represents another muscle-sparing alternative based on the same vascular pedicle, offering reduced donor-site morbidity for selected arm and axillary defects. However, TDAP flap dissection is technically demanding and requires considerable experience, and in contaminated defects, high-energy trauma, or cases requiring substantial tissue volume, the pedicled LD flap remains a more reliable option [19]. The circumflex scapular artery perforator flap, although capable of providing thin and pliable coverage for defects at the forearm and hand level, remains limited in elbow and arm reconstruction due to the requirement for microvascular anastomosis [20]. Similarly, for more distal defects of the hand and digits, local and regional perforator flaps such as the dorsal metacarpal artery flap, reverse radial forearm flap, and homodigital island flaps offer reliable alternatives tailored to the specific defect characteristics [21]. In these non-composite scenarios, mobilization of the latissimus dorsi muscle may represent an unnecessary sacrifice of a functional donor muscle. The pedicled LD flap is therefore more appropriately considered for extensive, complex, or composite defects requiring substantial tissue volume, durable soft-tissue coverage, or simultaneous functional restoration. In traumatic cases, concomitant fractures frequently necessitate internal fixation, and subsequent hardware exposure or wound breakdown over implants is a well-recognized complication requiring flap coverage. The pedicled LD flap provides robust, well-vascularized coverage over exposed hardware, facilitating fracture healing and reducing the risk of implant-related infection. Although local flaps can be used for small- to medium-sized defects, their limited arc of rotation and insufficient tissue volume make them unreliable for larger defects. Free flaps have significantly expanded reconstructive possibilities by enabling tissue transfer from distant sites. Nevertheless, in cases where recipient vessels are located within the zone of trauma, the reliability of microvascular anastomoses is reduced, and when suitable vessels are only available in distant regions, the use of vein grafts may become necessary [14,22]. This not only prolongs operative time but also increases the risk of complications.

Despite significant advances in reconstructive surgery, the pedicled latissimus dorsi (LD) flap remains one of the cornerstone options for the reconstruction of arm and elbow defects. Complex soft-tissue injuries of the upper extremity are characterized by extensive tissue loss, exposure of bone, joints, tendons, and neurovascular structures, as well as contamination and frequently associated vascular injury. These injuries most commonly result from high-energy trauma, electrical burns, or oncologic resections and are often accompanied by prolonged hospital stays, repeated debridements, an increased risk of infection, and a substantial threat of limb loss. In such scenarios, providing reliable, well-vascularized tissue coverage is crucial not only for defect closure but also for preventing amputation and preserving functional outcomes [23].

In this context, the pedicled latissimus dorsi (LD) muscle flap offers several advantages over free flaps in selected scenarios: it does not require microvascular anastomosis, shortens operative time, and provides more dependable outcomes in cases with compromised recipient vessels [24]. The absence of microvascular anastomosis is particularly beneficial in polytrauma patients with hemodynamic instability or significant comorbidities, in cases where recipient vessels lie within the zone of injury, and in institutions where microsurgical expertise is limited. This eliminates the risk of anastomotic thrombosis and associated flap loss. However, this advantage must be weighed against the limited reach and versatility compared to free tissue transfer, and in centers with established microsurgical capabilities, the choice between pedicled and free LD should be individualized based on defect location, size, and patient factors. It is particularly advantageous in the reconstruction of large and complex defects around the elbow and arm due to its substantial tissue volume and reliable arc of rotation. Although the radial forearm flap provides thin and pliable tissue, it is associated with considerable donor-site morbidity. The lateral arm flap, on the other hand, results in lower donor-site morbidity but is limited to the reconstruction of relatively small defects [25,26]. In contrast, the pedicled LD flap stands out as a more reliable option for the reconstruction of extensive and deep defects in these regions.

The role of the pedicled latissimus dorsi (LD) flap in upper extremity reconstruction is not limited to providing soft-tissue coverage; it also contributes to the preservation and, in selected cases, restoration of elbow flexion. Owing to its substantial muscle bulk and strong contractile capacity, the latissimus dorsi can be utilized as a functional muscle transfer to support elbow flexion. This is particularly relevant in cases involving loss of arm flexor muscles (biceps brachii and brachialis), destruction of the anterior compartment, or extensive soft-tissue defects. In such scenarios, the pedicled LD flap facilitates the restoration of elbow flexion, thereby enabling the functional use of the extremity in daily activities. Previous studies have demonstrated that pedicled latissimus dorsi transfer can improve elbow flexion strength and overall functional outcomes [27,28]. Azab and Alsabbahi evaluated 13 cases of bipolar LD transfer for elbow flexion restoration in traumatic brachial plexus injury and reported favorable functional results [29]. Similarly, Alshammari et al. described successful elbow flexion reconstruction using LD muscle transfer following road traffic accidents [30]. These reports support the reliability of pedicled LD transfer for functional elbow reconstruction. In our cohort, we employed a unipolar technique by preserving the humeral insertion of the latissimus dorsi and attaching the distal end of the muscle to the distal biceps tendon or, when the biceps tendon was unavailable, to the radius using a pseudotendon construct. Functional elbow flexion (≥90°) was achieved in 9 of 12 patients (75%), and antigravity strength was observed in 10 of 12 patients (83.3%). These results are consistent with the outcomes reported in the literature, where functional flexion has been achieved in 60–80% and antigravity strength in up to 87% of patients following pedicled LD transfer [10]. Although bipolar transfer has traditionally been advocated for providing a more direct line of pull and additional shoulder stabilization, several studies have shown that unipolar LD transfer can achieve comparable functional outcomes while offering the advantages of reduced operative time, less pedicle manipulation, and a lower risk of neurovascular compromise, hematoma, and thoracodorsal nerve injury [9,31]. This supports our choice of the unipolar technique, particularly in patients with extensive axillary scarring, where bipolar mobilization would pose an additional risk to the thoracodorsal pedicle.

Several alternative options exist for elbow flexion restoration and should be considered in the reconstructive algorithm [10]. These include bipolar transfer of the pectoralis major (Clark or Seddon procedure), Steindler flexorplasty (proximal advancement of the forearm flexor–pronator mass), free functioning gracilis muscle transfer with nerve coaptation (particularly useful in brachial plexus injuries), triceps-to-biceps transfer, and nerve transfers such as the Oberlin transfer or double fascicular nerve transfer [10,31]. Each of these techniques has specific advantages and limitations. The pedicled LD transfer is distinguished by its large muscle bulk, reliable vascular supply, and the ability to simultaneously address soft-tissue defects, which separates it from isolated tendon or nerve transfer techniques. In contrast, free gracilis transfer offers superior reach for more distal defects but requires microsurgical expertise and suitable recipient vessels [10]. The choice of technique should be tailored to the individual patient based on the level of injury, availability of donor muscles, nerve status, and the need for simultaneous soft-tissue coverage.

Another important goal of reconstruction in upper extremity trauma is to preserve functional bone length as much as possible to allow for prosthetic use. In severe injuries requiring shoulder disarticulation, preservation of the humeral stump is of critical importance for prosthetic adaptation and functional rehabilitation. Maintaining an adequately long humeral stump enhances prosthetic stability and contributes to greater independence in activities of daily living. Therefore, in suitable cases, preservation of the humeral stump combined with coverage using well-vascularized soft tissue is considered one of the key objectives of upper extremity reconstruction [16,32].

Another important advantage of the pedicled latissimus dorsi (LD) flap is its ability to be combined with the serratus anterior muscle when needed. These combined flaps, based on the thoracodorsal vascular system, can expand the reconstructive options by providing additional tissue in cases of circumferential or very large defects [33].

In terms of complications, the pedicled latissimus dorsi (LD) flap is generally considered a reliable technique. In our series, no total flap loss was observed, and the overall complication rate was comparable to or lower than those reported in the literature. Stevanovic et al. reported a distal necrosis rate of 19%, while Hacquebord et al. reported a rate of 17% [24,34]. In comparison, partial flap necrosis occurred in only one patient (3.8%) in our cohort, which may reflect careful patient selection and meticulous surgical technique. Donor-site seroma (11.5%) and wound dehiscence (7.7%) in our series are at the lower end of the range reported across the literature, where seroma incidence after LD flap harvest has been reported to range from approximately 6% to 79% [35,36]. We attribute this relatively low seroma rate to several intraoperative technical refinements. First, interrupted quilting sutures were routinely used to obliterate the dead space between the subcutaneous skin flaps and the underlying chest wall. This technique, widely validated in the breast reconstruction literature, has been shown to significantly reduce seroma incidence, seroma volume, and the duration of postoperative drainage [35,37,38]. Second, electrocautery was used at the lowest effective power setting, and intermittent saline irrigation was performed during its use to dissipate heat and minimize thermal injury to the surrounding fascia and subcutaneous tissues. Thermal injury has been identified as an important driver of seroma formation, and several comparative studies have demonstrated that reducing electrocautery-related tissue trauma is associated with lower seroma rates [39,40]. These simple yet reproducible technical measures, combined with standard closed-suction drainage, appear to contribute meaningfully to minimizing donor-site morbidity. The comprehensive systematic review by García-García et al. [41], which compiled 188 articles on all LD flap applications, confirmed that the pedicled LD flap remains predominantly indicated for anatomically adjacent defects such as the shoulder, arm, and elbow, supporting our use of this technique in the present cohort. Most of these complications can be managed with conservative measures or minor surgical interventions, and donor-site morbidity generally remains at an acceptable level.

The versatility of the latissimus dorsi flap across different reconstructive scenarios has been well documented in recent literature. Pinto et al. reviewed microsurgical reconstructive options, including pedicled LD flaps, for post-oncological upper limb defects and highlighted the importance of tailoring flap selection to the specific anatomical district [18]. Similarly, the LD flap has been extensively utilized in breast reconstruction, where technical refinements such as the ergonomic fat-augmented LD (FALD) flap described by Longo et al. have demonstrated superior aesthetic outcomes with vertical skin paddle orientation [42]. D’Orsi et al. further confirmed the long-term morphological stability of this technique, showing significantly higher breast projection in the ergonomic group compared with the traditional approach [43]. In the context of upper extremity necrotizing soft tissue infections, recent reviews have also emphasized the role of the LD flap as a reliable coverage option for large, complex wounds following extensive debridement [44]. Filigheddu et al. further demonstrated the reliability of the free LD flap for circumferential lower limb soft tissue defects following high-energy trauma, reporting no major complications and satisfactory functional recovery [45]. In the specific setting of electrical burn injuries, which constituted the majority of our cohort, Ziegler et al. highlighted the particular challenges posed by vascular damage and hypercoagulability, noting that careful assessment of recipient vessels is essential and that the pedicled LD flap may offer a distinct advantage by circumventing the need for microvascular anastomosis in such compromised tissue beds [46]. These studies collectively underscore the adaptability of the LD flap across diverse clinical scenarios, from oncological and traumatic reconstruction to aesthetic and functional applications. Additionally, Stumpfe et al. demonstrated in a prospective study of 94 patients that free muscle flaps, including the LD, when transferred without neurotization, result in profound and persistent sensory deficits, suggesting that fascio-cutaneous flaps with nerve coaptation should be considered in functionally critical regions where protective sensation is paramount [47]. This finding supports our approach of reserving the LD flap primarily for complex defects requiring volume and functional restoration rather than sensate coverage.

From a personalized medicine perspective, the present study highlights several key elements of individualized reconstructive planning. The choice of the pedicled LD flap itself was a patient-specific decision, favored over free tissue transfer or local flaps based on defect complexity, recipient vessel status, and the need for simultaneous functional restoration. Flap design (musculocutaneous vs. muscle-only), nerve management (preservation vs. transection of the thoracodorsal nerve), and postoperative rehabilitation were each tailored to the individual patient’s reconstructive goal and functional expectations. This level of treatment individualization reflects the growing recognition that optimal outcomes in reconstructive surgery depend on tailoring the entire care pathway to the unique needs of each patient.

This study has several limitations that should be acknowledged. First, the retrospective single-center design inherently limits the level of evidence. Second, the sample size is relatively small (*n* = 26), and the cohort is heterogeneous, encompassing different reconstruction goals, which limits the ability to draw definitive conclusions about any single indication. Third, the absence of a control group or comparison with alternative reconstructive techniques precludes a comparative assessment of the pedicled LD flap against other options. Fourth, and most importantly, standardized objective functional outcome measures were not systematically collected. Formal goniometric range of motion measurements, manual muscle strength grading, validated upper-limb outcome instruments such as the Disabilities of the Arm, Shoulder and Hand (DASH) score, and patient-reported outcome measures were not prospectively recorded. The functional observations presented in this study are therefore based on retrospective clinical documentation of activities of daily living and approximate estimates of elbow flexion range, rather than on objective measurements. Accordingly, the conclusions regarding functional restoration, particularly for elbow flexion, should be interpreted with caution. Fifth, the inherent limitations of the pedicled LD flap itself should be considered: its arc of rotation restricts reliable reach to the shoulder, arm, and elbow regions. Additionally, factors related to donor-site morbidity, including potential shoulder weakness in adduction and extension, seroma formation, and aesthetic concerns at the harvest site, are recognized drawbacks. The transferred LD may undergo variable degrees of atrophy over time, particularly when not reinnervated, potentially affecting long-term functional outcomes. Future prospective studies with larger sample sizes and standardized functional assessments, including DASH scores and goniometric measurements, are needed to further validate the efficacy of the pedicled LD flap in this setting.

## 5. Conclusions

The present study demonstrates that the pedicled latissimus dorsi (LD) flap is a reliable and versatile option for the reconstruction of complex arm and elbow soft-tissue defects. Particularly in cases following electrical burns and high-energy trauma, where major neurovascular structures are exposed, the well-vascularized tissue provided by the flap enables limb salvage and facilitates the achievement of the intended reconstructive goals. The individualized, goal-directed approach adopted in this study—in which flap design, nerve management, and rehabilitation were tailored to each patient’s specific defect characteristics and functional expectations—underscores the relevance of personalized medicine principles in reconstructive surgery.

The absence of a need for microvascular anastomosis, its applicability independent of recipient vessels within the zone of trauma, and its ability to provide substantial tissue volume make the pedicled LD flap a practical and dependable reconstructive method, especially in contaminated and advanced defects. Although free flaps and perforator-based alternatives represent valuable options in selected cases, the pedicled LD flap continues to maintain its relevance in arm and elbow reconstruction due to its technical reliability, acceptable donor-site morbidity, and contribution to limb preservation.

Furthermore, by allowing preservation of the humeral stump in appropriate cases, it supports the maintenance of an amputation level suitable for prosthetic use, thereby enhancing functional rehabilitation. From this perspective, the pedicled LD flap should be considered a versatile surgical option that provides both limb-salvage and coverage-based reconstruction in complex traumatic upper extremity injuries. However, the functional outcomes reported in this study are based on clinical observation rather than standardized assessments, and prospective studies incorporating validated outcome measures are warranted to strengthen these conclusions.

## Figures and Tables

**Table 1:** Demographic characteristics and etiology of injury.

	*n* (%)
Total number of patients	26
Male	22 (84.6%)
Female	4 (15.4%)
Age range	8–35 years
Etiology	
Electrical burn	24 (92.3%)
Traffic accident	2 (7.7%)

**Table 2:** Reconstruction goals.

Reconstruction Goal	*n* (%)
Elbow flexion restoration with brachial artery and median nerve repair + soft-tissue coverage	2 (7.7%)
Elbow flexion restoration with brachial artery repair + soft-tissue coverage	2 (7.7%)
Elbow flexion restoration + soft-tissue coverage	7 (26.9%)
Elbow flexion restoration only	1 (3.8%)
Preservation of the humeral stump for prosthetic use	14 (53.8%)
Total	26 (100%)

**Table 3:** Flap outcomes and postoperative complications.

Outcome/Complication	*n* (%)
Total flap loss	0 (0%)
Partial flap necrosis	1 (3.8%)
Donor-site wound dehiscence	2 (7.7%)
Donor-site seroma	3 (11.5%)
Donor-site hematoma	0 (0%)
Recipient-site hematoma	1 (3.8%)
Need for amputation, stump revision, or shoulder disarticulation	0 (0%)
Total number of flaps	26

## Data Availability

The original contributions presented in this study are included in the article. Further inquiries can be directed to the corresponding author.

## Abbreviations

The following abbreviations are used in this manuscript:

LD	Latissimus dorsi
IUCAP	Inferior ulnar collateral artery perforator
SUCAP	Superior ulnar collateral artery perforator
BAP	Brachial artery perforator
RRAP	Radial recurrent artery perforator
RCAP	Radial collateral artery perforator
TDAP	Thoracodorsal artery perforator
DASH	Disabilities of the arm, shoulder, and hand
FALD	Fat-augmented latissimus dorsi
